# Bayesian graphical models for modern biological applications

**DOI:** 10.1007/s10260-021-00572-8

**Published:** 2021-05-27

**Authors:** Yang Ni, Veerabhadran Baladandayuthapani, Marina Vannucci, Francesco C. Stingo

**Affiliations:** 1 Department of Statistics, Texas A&M University, College Station, USA; 2 Department of Biostatistics, University of Michigan, Ann Arbor, USA; 3 Department of Statistics, Rice University, Houston, USA; 4 Department of Statistics, Computer Science, Applications “G. Parenti”, The University of Florence, Florence, Italy

**Keywords:** Graphical models, Bayesian methods, Complex data, Genomics, Neuroimaging

## Abstract

Graphical models are powerful tools that are regularly used to investigate complex dependence structures in high-throughput biomedical datasets. They allow for holistic, systems-level view of the various biological processes, for intuitive and rigorous understanding and interpretations. In the context of large networks, Bayesian approaches are particularly suitable because it encourages sparsity of the graphs, incorporate prior information, and most importantly account for uncertainty in the graph structure. These features are particularly important in applications with limited sample size, including genomics and imaging studies. In this paper, we review several recently developed techniques for the analysis of large networks under non-standard settings, including but not limited to, multiple graphs for data observed from multiple related subgroups, graphical regression approaches used for the analysis of networks that change with covariates, and other complex sampling and structural settings. We also illustrate the practical utility of some of these methods using examples in cancer genomics and neuroimaging.

## Introduction

Graphical models have been widely applied to describe the conditional dependence structure of a *p*-dimensional random vector; a graphical model is a pair consisting of a graph *G* and an associated probability distribution respecting the conditional independence encoded by *G*. Graphical models have been extensively studied in the literature for both directed (Friedman et al. 2000; Spirtes et al. 2000; Geiger and Heckerman 2002; Shojaie and Michailidis 2010; Stingo et al. 2010) and undirected graphs (Dobra et al. 2004; Meinshausen and Bühlmann 2006; Yuan and Lin 2007; Banerjee et al. 2008; Friedman et al. 2008; Carvalho and Scott 2009; Kundu et al. 2013; Stingo and Marchetti 2015). In this paper we review some recent Bayesian techniques developed to estimate large graphical models for complex data structures, motivated by applications in biology and medicine. Our focus is on non-standard settings with particular interest in heterogeneous data, integrative graphical models for multiple related subgroups, and multi-dimensional graphical models for data measured with covariates and along multiple axes/dimensions.

In the context of large networks, Bayesian approaches are particularly suitable because prior distributions can be used both to encourage sparsity of the graphs, which is a realistic assumption for many real-world applications including inference of biological networks, and to incorporate prior information in the inferential process. Moreover, Bayesian approaches allow us to naturally account for uncertainty in the graph structure; graph uncertainty is especially important in the context of high-dimensional complex data, since with a limited sample size, several graphs may explain the data equally well and hence point estimators are often not adequate.

Many of the motivating applications of the methodology presented in this review come from cancer genomics, although the methodology is general and applicable to diverse contexts. Cancer is a set of diseases characterized by coordinated genomic alterations, the complexity of which is defined at multiple levels of cellular and molecular organization (Hanahan and Weinberg 2011). The application of Bayesian graphical models to cancer genomics as well as other disease types hinges on the ability of these methods to learn biological networks that describe the various complex regulatory and associations patterns in molecular units (genes or proteins) across different organs and organ systems (Iyengar et al. 2015). The overarching goal of the methodology discussed in the following sections is to provide an enhanced understanding of the biological mechanisms underlying the disease of interest.

A key task to this end is to develop flexible and efficient quantitative models for the analysis of dependence structures of these high-throughput assays. Several approaches have been developed for the analysis of genomic or proteomic networks, including co-expression, gene regulatory, and protein interaction networks (Friedman 2004; Dobra et al. 2004; Mukherjee and Speed 2008; Stingo et al. 2010; Telesca et al. 2012). However, these methods lack the ability to analyze heterogeneous populations, characterized, for example, by networks that change with respect to covariates. More generally, the methodology we present for the analysis of complex networks directly applies to other scientific applications such as the analysis of disease subgroups, experiments performed under different conditions, or even settings that go beyond biology and medicine.

We do not aim to provide a comprehensive review of standard graphical models with e.g., the independent and identically distributed (iid) assumption; nor do we attempt to cover different learning strategies (algorithmic versus probabilistic). Rather we focus on reviewing recently developed Bayesian probabilistic graphical models for large-scale biological networks under non-iid settings with the hope to stimulate future research in this exciting area. For broader dissemination, we also make available the codes for the multiple graphical model^1^ and the graphical regression model,^2^ which generate the results in Sects. 3.2 and 4.2.

The rest of the paper is organized as follows: basic concepts of Bayesian inference of graphical models are presented in Sect. 2. In Sect. 3 we describe models for the analysis of multiple related networks, one for each of the sub-population. We discuss approaches for networks that change with covariates in in Sect. 4, and provide an overview of methods for other complex data and network structures in Sect. 5. We conclude with a brief discussion in Sect. 6.

## Basic concepts in graphical modeling

In this section we provide some background material concerning undirected and directed graphical models. More information on graphs and graphical models can be found in Lauritzen (1996b). We also briefly describe some recent techniques developed for the analysis of homogeneous populations (single networks).

### Undirected Gaussian graphical models

Let $G=(V,E)$ be a graph defined by a set of nodes, $j \in V$ and a set of edges $(i,j) \in E$ joining pairs of nodes $i,j \in V$, and let $Y=(Y_{j})_{j \in V}$ be a $p \times 1$ random vector indexed by the finite set *V* with $p=|V|$. A graph, associated to a random vector *Y*, is generally used to represent conditional independence structures under suitable Markov properties. Typically, missing edges in *G* correspond to conditional independencies for the joint distribution of *Y*. An *undirected Gaussian graphical model (GGM)* is a family of multivariate normal distributions for *p* variables $Y = (Y_1, \dots , Y_p)^{ \mathrm {\scriptscriptstyle T} }\sim N_p(\mu , \varSigma )$ with mean $\mu$, and positive definite covariance matrix $\varSigma$ defined by a set of zero restrictions $\omega _{ij} = 0$ on the elements of concentration matrix $\varOmega = \varSigma ^{-1} = (\omega _{ij})$. Each constrain $\omega _{ij} = 0$ is equivalent to a conditional independence of $Y_i$ and $Y_j$ given the remaining variables, written as $Y_i \, \perp \perp Y_j \mid Y_{V\setminus ij}$. In fact, in a Gaussian model conditional independence is equivalent to zero partial correlation between $Y_i$ and $Y_j$ given the rest

$$\begin{aligned} Y_i \, \perp \perp Y_j \mid Y_{V\setminus ij} \iff \rho _{ij.V\setminus ij} = 0 \iff \omega _{ij} = 0. \end{aligned}$$

The likelihood function of a random sample of *n* independent and identically distributed (iid) observations $Y^{(1)}, \dots , Y^{(n)}$ from $N_p(0, \varOmega )$ is

1 $$\begin{aligned} L(\varOmega |S) \propto (\det \varOmega )^{n/2}\exp \{\textstyle \frac{1}{2} \mathrm {tr}(\varOmega S)\} , \end{aligned}$$

where $\varOmega$ is in the parameter space

2 $$\begin{aligned} P_G = \{ \varOmega \text { positive definite }p\times p\text { matrix} : \omega _{ij} = 0 \text { whenever } \{i, j\} \not \in E\} \end{aligned}$$

and $S = \sum _{l=1}^n Y^{(l)}(Y^{(l)})^{ \mathrm {\scriptscriptstyle T} }$ is the sample sum-of-products matrix. The parameter space $P_G$ has a complex structure, being the cone of positive-definite matrices with zero-patterns compatible with the missing edges in *G*.

### Bayesian inference of undirected GGMs

In this section we briefly review Bayesian approaches for inference on both the graph structure *G* and precision matrix $\varOmega$. A fully Bayesian approach provides a clear measure of uncertainty on the estimated network structures. For the special case of decomposable graphs, efficient algorithm based on hyper-inverse Wishart priors can be implemented (Roverato 2000). In this context, marginal likelihoods of the graph can be calculated in closed form (Clyde and George 2004). Jones et al. (2005) proposed an approach for graph selection for both decomposable and nondecomposable high-dimensional models; computations for the nondecomposable case were found to be much more cumbersome. Alternative stochastic algorithms for inference of decomposable models include the feature-inclusion stochastic search algorithm of Scott and Carvalho (2008); this approach uses online estimates of edge-inclusion probabilities and scales to larger dimensions reasonably well in comparison with Markov chain Monte Carlo (MCMC) algorithms.

Decomposable graphs are a small subset of all possible graphs, and are not appropriate in many applied settings. From a computational perspective, the key difference between decomposable and nondecomposable models hinges on the calculation of the normalizing constant of the marginal likelihoods. For the decomposable case, it can be exactly calculated; whereas for nondecomposable graphs the same calculation relies on expensive numerical approximations. Many popular approaches for nondecomposable graphs are based on the *G*-Wishart prior for precision matrices (Atay-Kayis and Massam 2005); conditional on a given graph *G*, this prior imposes that the elements of the precision matrix that correspond to missing edges are set exactly to zero. Dobra et al. (2011) proposed an efficient Bayesian sampler that avoids the direct calculation of posterior normalizing constants. Wang and Li (2012) proposed an exchange algorithm based on *G*-Wishart priors that bypasses the calculation of prior normalizing constants and it is overall computationally more efficient than the one proposed by Dobra et al. (2011). Building upon the decomposable Gaussian graphical model framework, Stingo and Marchetti (2015) proposed a computationally efficient approach that exploits graph theory results for local updates that facilitate fast exploration of the space of all nondecomposable graphs. Mohammadi and Wit (2015) developed a computationally efficient trans-dimensional MCMC algorithm based on continuous-time birth-death processes that performs comparatively very well with respect to alternative Bayesian approaches in terms of computing time and graph reconstruction, particularly for large graphs; this algorithm is part of the R package BDgraph (Mohammadi and Wit 2019).

Methods based on priors alternative to the *G*-Wishart prior have been developed to overcome the computational burden that comes with this approach. Continuous shrinkage priors are a viable alternative that results in algorithms for posterior inference which are more efficient and have greater scalability. Continuous shrinkage priors such as scale mixture of normal distributions (Carvalho et al. 2010; Griffin et al. 2010) and the spike-and-slab prior (George and McCulloch 1993), have been extensively studied for variable selection in regression models, and recently used in estimating covariance and precision matrices (Wang 2012). Methods that are suited for the analysis of large undirected graphs include stochastic search structure learning algorithm of Wang (2015). This method is based on continuous shrinkage priors indexed by binary indicators that are basically the elements of the adjacency matrix of the graph; the companion algorithm exploits efficient block updates of the network parameters and result in relatively fast computation.

### Directed acyclic graphs

A *directed acyclic graph* (DAG), also called a *Bayesian network*, $G= (V,E)$ consists of a set $V=(1,2,\dots ,p)$ of nodes, representing random variables $\{Y_1,Y_2,\dots ,Y_p\}$, as in the undirected case, and a set $E\subseteq \{(i,j): i,j\in V\}$ of directed edges, representing the dependencies between the nodes. Denote a directed edge from *i* to *j* by $i\rightarrow j$ where *i* is a parent of *j*. The set of all the parents of *j* is denoted by $\textit{pa(j)}$. The absence of edges represents conditional independence assumptions. We assume that there are no cycles in the graph (i.e., there is no path that goes back to the starting node), which allows for factorization of the joint distribution as the product of the conditional distributions of each node given its parents:

3 $$\begin{aligned} P(Y_1,\dots ,Y_p)=\prod _{g=1}^p P(Y_g|Y_{pa(g)}), \end{aligned}$$

where $Y_{pa(g)}=\{Y_j:j\in pa(g)\}$. Without loss of generality, the ordering is defined as $\{1,2,\dots ,p\}$, which can be obtained through prior knowledge such as known reference biological pathways, for example. Define $[g-]$ to be the set $\{1,2,\dots ,g-1\}$ and $y_{[g-]}$ to be $\{y_i:i\in [g-]\}$. Each conditional distribution in the product term of equation (3) can be expressed by the following system of recursive regressions:

4 $$\begin{aligned} Y_g = f_g(Y_{[g-]})+\epsilon _g, \,\,\, g=1,2,\dots ,p, \end{aligned}$$

where $f_g(Y_{[g-]})$ is the predictor function and $\epsilon _g$ is the error term; if the error terms are iid and normally distributed, $\epsilon _g \sim N(0,\lambda _g^{-1})$, and $f_g(\cdot )$ is the classical linear predictor, then the joint distribution of *Y* is *p*-dimensional multivariate Gaussian.

Note that if an ordering of the nodes is not specified, we cannot distinguish between two Gaussian DAGs that belong to the same *Markov equivalence class*. DAGs within this class have the same skeleton and v-structures, and they represent the same conditional independence structure (Lauritzen 1996b). Given an observational dataset, two Gaussian DAGs belonging to the same Markov equivalence class will have the same likelihood function and cannot be distinguished without further assumptions; throughout this paper, we will assume a known node ordering, given which all Markov equivalence classes have size one.

### Bayesian inference of directed acyclic graphs

If there is a known ordering of the nodes, DAGs can be framed as a set of independent regression models. In this setting techniques developed for variable selection, such as the spike-and-slab prior (George and McCulloch 1993), can be easily adapted to infer graph structures. For example, Stingo et al. (2010) developed a framework for inference of miRNA regulatory networks as DAGs. The ordering of the variables is determined by the biological role of the observed variables. This framework can be extended to account for non-linear association, as proposed by Ni et al. (2015); each conditional distribution was represented by a semi-parametric regression model based on penalized splines and variable selection priors that can discriminate linear and non-linear associations. Alternative approaches to spike-and-slab priors are also possible, one example is the objective Bayesian approach, based on non-local priors, proposed by Altomare et al. (2013).

If the ordering of the variables in unknown, two Gaussian DAGs that belong to the same Markov equivalence class can not be distinguished based on observational data. In this setting DAGs can be partitioned into Markov equivalence classes, and each class can be represented by a chain graph called *Essential Graph* (EG) (Andersson et al. 1997) or *Completed Partially Directed Acyclic Graph* (CPDAG) (Chickering 2002). Castelletti et al. (2018) proposed an approach for model selection of EGs/CPDAGs using a method based on the fractional Bayes factor; notably, this approach results in closed form expression for the marginal likelihood of an EG/CPDAG that can be used for model selection.

## Bayesian multiple graphs

Consider a dataset of gene expression measurements collected from a set of subjects affected by a given disease, and assume that these patients can be grouped by disease stage. For many diseases, the biological network representing important cellular functions may evolve with disease stage. Each subgroup of patients should be then characterized by a different gene network. In the example above and in many other scenarios, samples can be naturally divided into homogeneous subgroups. If we can reasonably assume that the sampling model of each subgroup can be represented by a graphical model, then methods for multiple graphical models are an appropriate choice for data analysis. In such cases, if we infer a single network using the entire data set as the basis for inference we may identify spurious relationships, results may not be easily interpreted, and we may also miss important connections present in many subgroups but missing in few others. Alternatively, we may perform an analysis of each subgroup separately; this approach considerably reduces the sample size, as in many real world scenarios we may end up with very small subgroups.

The approaches we discuss in this section are designed to analyze multiple directed or undirected networks in settings where some networks may be totally different, while others may have a similar structure. We focus on the approach proposed by Peterson et al. (2015). This approach is based on Markov random field (MRF) priors and infers a different network for each subgroup but it encourages some networks to be similar when supported by the data.

### Approaches based on Markov random field priors

We focus on Bayesian approaches to the problem of multiple undirected network inference based on MRF priors. These priors link the estimation of the group specific graphs encouraging common structures. In practice, the inclusion of an edge in the network of a given group is encouraged if the same connection is present in the graphs of related groups. A key aspect of this methodology is the absence of the otherwise common assumption in approaches based on penalized likelihoods, e.g., Danaher et al. (2014), that all subgroups are related. Unlike alternative approaches in the frequentist framework (Pierson et al. 2015; Saegusa and Shojaie 2016), which require a preliminary step to learn which subgroups are related, the approach proposed by Peterson et al. (2015) learns both the within-group and cross-group relationships. Another key difference is that, even though penalization based approaches can be applied to problems of higher dimensions, they provide only point estimates of large networks, which are often unstable given limited sample sizes. By taking a Bayesian approach, it is possible to quantify uncertainty in the network estimates.

The basic model setup can be summarized as follow. Let *K* be the number of sample subgroups, and ${\mathbf {Y}}_k$ be the $n_k \times p$ matrix of observed data for sample subgroup *k*, where $k = 1, 2, \ldots , K$. The same *p* random variables are observed across all subgroups; the sample sizes $n_k$ do not need to be identical. Within each subgroup, observations are iid, and under the normality assumption the contribution to the likelihood of subject *i* in group *k* is ${\mathbf {y}}_{k,i} \sim {\mathcal {N}}(\varvec{\mu }_k,\mathbf {\varOmega }^{-1}_k), i = 1, \ldots , n_k$, where $\varvec{\mu }_k \in {\mathbb {R}}^p$ is the vector of expected values for subgroup *k*, and $\mathbf {\varOmega }_k$ is the precision matrix for the same subgroup constrained by a graph $G_k$ specific to that subgroup, with a generic element $g_{k,ij}$ indicating the inclusion of edge (*i*, *j*) in $G_k$; $\varvec{\mu }_k$, $\mathbf {\varOmega }_k$, and $G_k$ are the subgroup specific model parameters.

At the cornerstone of this methodology is an MRF that links all *K* networks. This prior is designed to share information across subgroups, when appropriate, and to incorporate relevant prior knowledge, when available. In this context, an MRF is used as the prior distribution of the indicators of edge inclusion $g_{k,ij}$. For each edge (*i*, *j*), we define the $K \times 1$ binary vector $\mathbf{{g_{ij}}}=(g_{1, ij}, \ldots , g_{K, i j})^T$ where $1\le i < j \le p$, and impose a MRF prior distribution such as

$$\begin{aligned} p(\mathbf{{g_{ij}}}|\nu _{ij}, \varTheta )=C(\nu _{ij}, \varTheta )^{-1} \exp (\nu _{ij} \mathbf{{1}}^T \mathbf{{g_{ij}}}+\mathbf{{g_{ij}}}^T \varTheta \mathbf{{g_{ij}}}), \end{aligned}$$

where $\nu _{ij}$ is connected to baseline prior probability of selecting edge (*i*, *j*), $\varTheta$ is a $K\times K$ symmetric matrix representing pairwise between-group associations, and $\mathbf{{1}}$ is the *K*-dimensional vector of ones. The off-diagonal elements of $\varTheta$, $\theta _{km}$, are the parameters that connect the *K* networks since a non-zero $\theta _{km}$ implies that groups *k* and *m* share information; the posterior distribution of these parameters can be interpreted as a measure of relative network similarity across the groups. From a computational perspective, particular care is needed in dealing with the normalizing constant $C(\nu _{ij}, \varTheta )=\sum _{\mathbf{{g_{ij}}}\in \{0,1\}^K} \exp (\nu _{ij}{} \mathbf{{1}}^T\mathbf{{g_{ij}}}+\mathbf{{g_{ij}}}^T\varTheta \mathbf{{g_{ij}}})$. As long as the number of subgroups *K* is small or the parameters $\nu$ and $\varTheta$ are fixed to constant values, the computation of this constant is feasible; otherwise methods for doubly unknown normalizing constants need to be implemented (Møller et al. 2006; Stingo et al. 2011).

The joint prior on the graphs $(G_1, \ldots , G_K)$ is the product of the densities for each edge $p(G_1, \ldots , G_k|\mathbf{{\nu }}, \varTheta ) = \prod _{i<j} p(\mathbf{{g_{ij}}}|\nu _{ij}, \varTheta ),$ where $\mathbf{{\nu }}=\{\nu _{ij}|1\le i <j \le p\}$. Prior distributions on $\mathbf{{\nu }}$ and $\varTheta$ complete the prior specification. A prior on $\mathbf{{\nu }}$ controls the overall sparsity of the networks, and can be set to reduce false selection of edges (Scott and Berger 2010; Peterson et al. 2015). A prior on the $K \times K$ symmetric matrix $\varTheta$ characterizes the *a priori* similarity of the graphs between the subgroups. Specifically, each off-diagonal element $\theta _{km}$ represents the similarity between subgroup *k* and subgroup *m*. This prior can be defined to learn which groups are related (in terms of network structure), and if they are, how strong this similarity is. Peterson et al. (2015) proposed the following spike and slab prior on each $\theta _{km}$:

$$\begin{aligned} p(\theta _{km}|\gamma _{km})&=(1-\gamma _{km})\delta _0+\gamma _{km}\frac{\beta ^\alpha }{\varGamma (\alpha )}\theta _{km}^{\alpha -1}e^{-\beta \theta _{km}}, \end{aligned}$$

where $\alpha$ and $\beta$ are fixed hyper parameters, and the binary indicator $\gamma _{km}$ determines whether subgroups *k* and *m* have related network structure. The binary indicators $\gamma _{km}$’s follow independent Bernoulli priors. If $\gamma _{km}=0$, this prior does not encourage similarity (i.e., subgroups have different graph structures); if $\gamma _{km}=1$, this prior encourages borrowing strength between subgroups *k* and *m*. A Bernoulli prior is imposed on $\gamma _{km}\sim \text{ Bernoulli }(\psi )$.

Within this prior framework, we can easily incorporate prior knowledge on specific connections through the prior on $\varvec{\nu }$. Larger values of $\nu _{ij}$ give connection (*i*, *j*) higher probability to be selected *a priori*. For example, if $G_0 =(V,E_0)$ is a reference network whose connections we want to give higher prior probabilities, we can define a prior distribution on $q_{ij} = e^{\nu _{ij}}/(1+e^{\nu _{ij}})$, the logistic transformation of $\nu _{ij}$, such that

5 $$\begin{aligned} q_{ij} = {\left\{ \begin{array}{ll}\text {Beta}(1+c,1) \text { if } (i,j) \in E_0 \\ \text {Beta}(1,1+c) \text { if } (i,j) \notin E_0, \end{array}\right. } \end{aligned}$$

where $c > 0$. The corresponding prior on $\nu _{ij}$ can be written as

6 $$\begin{aligned} p(\nu _{ij}) = \frac{1}{B(a,b)}\cdot \frac{e^{a\nu _{ij}}}{(1+e^{\nu _{ij}})^{a+b}}, \end{aligned}$$

where $B(\cdot )$ represents the beta function. If no such prior knowledge is available, sparsity can be induced setting $q_{ij} \sim \text {Beta}(1,4)$ for all edges (*i*, *j*); a discussion of other relevant prior settings can be found in Peterson et al. (2015).

*Completing the model and computational aspects.* A conjugate multivariate normal prior on the vector $\varvec{\mu }_k$ is usually the default choice (Peterson et al. 2015). The prior on the precision matrices $\varOmega _k$ has important implications in terms of computation and then scalability. Two relevant options are available. Peterson et al. (2015) choose a *G*-Wishart distribution $\varOmega _k | G_k \sim W_G(b, {\mathbf {D}})$ (Dobra et al. 2011); this prior gives positive density to the cone of symmetric positive definite matrices $M^+$, with $\omega _{k,ij}$ exactly equal to zero for any edge $(i,j) \notin E_k$. This is a good modeling property; unfortunately, both the prior and posterior normalizing constants, needed to calculate the transition kernel of the companion MCMC algorithm, are intractable, and consequently this method does not scale well with the number of observed variables *p* (Peterson et al. 2015). Alternatively, Shaddox et al. (2018) formulate a method based on the continuous shrinkage prior for precision matrices proposed by Wang (2015). This continuous prior is defined by the product of $p(p-1)/2$ spike-and-slab mixture densities, corresponding to the off-diagonal elements, and *p* exponential densities, corresponding to the diagonal elements:

$$\begin{aligned} p(\varOmega _k|G_k)&\propto \prod _{i<j} N(\omega _{ij}|0, \upsilon _{g_{ij}}^2)\prod _i {\text {Exp}}\left( \omega _{ii}|\frac{\lambda }{2}\right) , \end{aligned}$$

where $\upsilon _{g_{ij}}^2=\upsilon _1^2$ if $g_{k,ij} =1$ , and $\upsilon _{g_{ij}}^2=\upsilon _0^2$ if $g_{k,ij}=0$; hyperparameters can be set such that only one component of the mixture is concentrated around zero (Wang 2015; Shaddox et al. 2018). The companion MCMC algorithm ensures that the sampled precision matrix belong to $M^+$, and can be used for the analysis of relatively large networks.

### Application of multiple graphical models to multiple myeloma genomics data

**Fig. 1:**
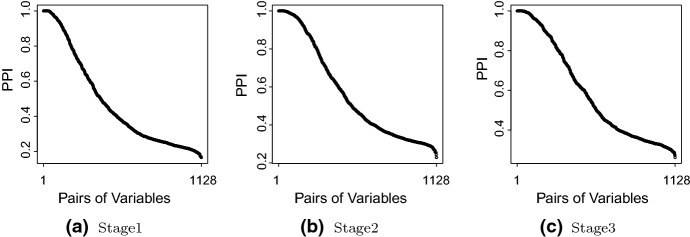
Posterior probability of inclusion (PPI)

We apply the multiple graphical model (Shaddox et al. 2018) to multiple myeloma gene expression data collected by the Multiple Myeloma Research Consortium (Chapman et al. 2011). Multiple myeloma is a late-stage malignancy of B cells in the bone marrow. We focus on the genes that are the core members of the five critical signaling pathways identified by previous multiple myeloma studies (Boyd et al. 2011): (1) Ras/Raf/MEK/MAPK pathway, (2) JAK/STAT3 pathway, (3) PI3K/AKT/mTOR pathway, (4) NF-$\kappa$B pathway and (5) WNT/$\beta$-catenin pathway. After removing samples with missing values, we have $n=154$ samples and $p=48$ genes. Alternatively, the missing data could have been imputed within the Bayesian framework using posterior predictive distribution if they are missing completely at random. According to the International Staging System (Greipp et al. 2005), multiple myeloma is classified into three stages by two important prognostic factors, serum beta-2 microglobulin (S$\beta _2$M) and serum albumin: stage I, S$\beta _2$M $<3.5$ mg/L and serum albumin $\ge 3.5$ g/dL; stage II, neither stage I nor III; and stage III, S$\beta _2$M $\ge 5.5$ mg/L. This application aims to construct stage-specific multiple myeloma gene co-expression networks. We run MCMC for 10,000 iterations with 5000 burn-in, which takes 0.6 hour. The hyperparameters are fixed at $\upsilon _0^2=0.0004$, $\upsilon _1^2=1$, $\lambda =1$, $\alpha =4$, $\beta =5$, $\psi =.9$, $a=1$, and $b=4$. In large scale inference, graph structure reconstruction is critical and challenging, particularly due to large number of parameters to be estimated (on the order of ${\mathcal {O}}(p^2)$). Furthermore, fully Bayesian approaches have the advantage of providing a clear measure of graph uncertainty. As shown in Fig. 1, we can learn the edge posterior probability of inclusion (PPI) for each group; we can identify which edges are supported by the data, and we can quantify our confidence in the inclusion of each edge into the selected graph. Alternatively, we could have selected the graph with the highest posterior probability; many graphs may have a similar posterior probability, making this second option for model selection less used in practice.

We used the posterior expected FDR to choose the probability cutoff for posterior probability of inclusion. Specifically, the posterior expected FDR of the multiple graphical model is defined as

$$\begin{aligned} E[\text{ FDR}_{c}|\text{ data}]=\frac{\sum _k\sum _{i<j}(1-p_{k,i,j})1(p_{k,i,j}> c)}{\sum _k\sum _{i<j}1(p_{k,i,j}> c)}, \end{aligned}$$

where $p_{k,i,j}=p(g_{k,i,j}=1|\varvec{Y}_1,\dots ,\varvec{Y}_K)$ is the posterior probability of edge inclusion. And the cutoff *c* is chosen to be $\min \{c| E[\text{ FDR}_{c}|\text{ data}]\le 0.01\}$. A similar procedure is used for graphical regression estimation in Sect. 4.2.

**Fig. 2:**
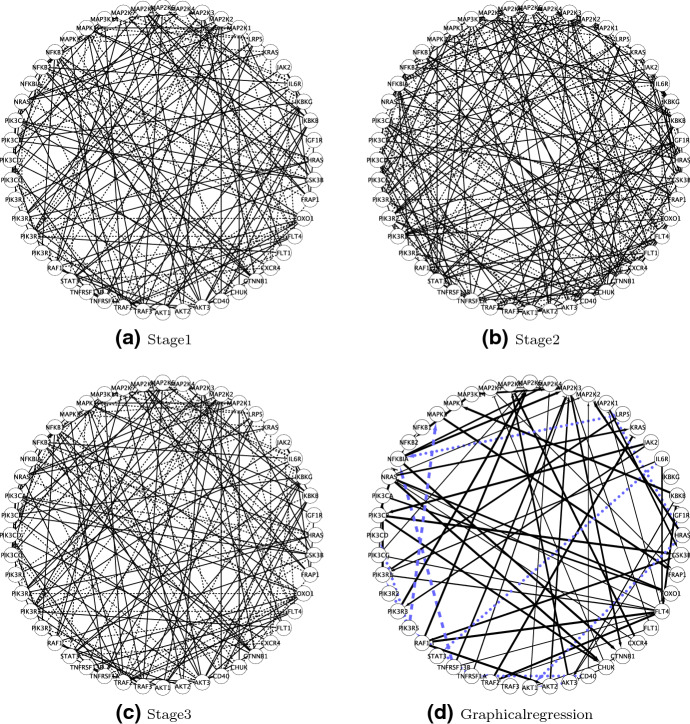
Multiple myeloma network analyses. Panels (a)-(c). The estimated stage-specific gene co-expression networks. The solid lines indicate positive partial correlations and the dashed lines indicate negative partial correlations. Panel (d). The estimated gene regulatory network from graphical regression integrating the prognostic factors: S$\beta _2$M and serum albumin. The solid lines with arrowheads indicate positive constant effects; solid lines with flat heads indicate negative constant effects; dashed lines indicate linearly varying effects; dotted lines indicate nonlinearly varying effects; the width of the solid line is proportional to the posterior probability of inclusion

The estimated stage-specific networks are shown in Fig. 2a–c with FDR controlled at 1%. They have 89, 136, and 119 edges. The estimated association across stages is $$ {\widehat{\varTheta }}=\left[ \begin{array}{ccc}1.00&{}0.30&{}0.39\\ 0.30&{}1.00&{}0.68\\ 0.39&{}0.68&{}1.00\end{array}\right] $$, which shows stages II and III have the greatest similarity in gene network structure. In addition, for comparison, we compute the network similarities based on two *ad hoc* metrics. The first metric is the Hamming distance $D_h(k,k')=\sum _{i,j}I({\hat{g}}_{k,i,j}\ne {\hat{g}}_{k',i,j})$ of estimated graphs between stages *k* and $k'$ where ${\hat{g}}_{k,i,j}=I(p(g_{k,i,j}=1|\varvec{Y}_1,\dots ,\varvec{Y}_K)>c)$ for some probability cutoff *c* (*c* is chosen to control FDR at 1% in this application). The pairwise hamming distances between stages are $D_h(1,2)= 177, D_h(1,3)= 174$, and $D_h(2,3)= 217$. Here, stages II and III have the largest distance. Note that the metric $D_h$ is based on marginal edge inclusion ${\hat{g}}_{k,i,j}$ whereas ${\widehat{\varTheta }}$ provides an overall/joint network similarity measure. Moreover, $D_h$ depends on the probability cutoff *c* used to obtain ${\hat{g}}_{k,i,j}$. The second metric is the $\ell _1$ distance $D_1(k,k')=\sum _{i,j}|p_{k,i,j}-p_{k',i,j}|$ of posterior edge inclusion probabilities $p_{k,i,j}=p(g_{k,i,j}=1|\varvec{Y}_1,\dots ,\varvec{Y}_K)$ between stages *k* and $k'$. The pairwise $\ell _1$ distances between stages are $D_1(1,2)= 310.45, D_1(1,3)= 319.22$, and $D_1(2,3)= 298.03$. Not relying on the probability cutoff (although still based on marginal, rather than joint, edge inclusion probabilities), $D_1$ agrees with ${\widehat{\varTheta }}$ that stages II and III have the greatest similarity.

### Extensions to dynamic graphical models for estimation of brain connectivity

Warnick et al. (2018) extended the work of Peterson et al. (2015) to a framework for the estimation of dynamic graphical models, with the specific purpose of studying dynamic brain connectivity based on fMRI data. Brain connectivity is defined as the set of correlations or causal relationships between brain regions that share similar temporal characteristics (Friston et al. 1994). Traditionally, brain network studies have assumed connectivity as spatially and temporally stationary, i.e. connectivity patterns are assumed not to change throughout the scan period. However, in practice, the interactions among brain regions may vary during an experiment. For example, different tasks, or fatigue, may trigger varying patterns of interactions among different brain regions. More recent approaches have regarded brain connectivity as *dynamic* over time. For example, Cribben et al. (2012) investigated greedy approaches that recursively estimate precision matrices using the graphical LASSO on finer partitions of the time course of the experiment and select the best resulting model based on BIC. The approach proposed by Warnick et al. (2018) directly estimates change points in the connectivity dynamics through a hidden Markov model (HMM) on the graphical network structures, therefore avoiding arbitrary partitions of the data into sliding windows.

Let $\varvec{Y}_{t}=(Y_{t1}, \ldots , Y_{tp})^\top$ be the vector of fMRI responses measured on a subject at *p* regions of interest (henceforth ROIs) at time *t*, for $t=1, \ldots , T$. In the following, we will refer to ROIs as macro-areas of the brain which comprise multiple voxels that covary in time. We start by assuming that the observed measurements can be modeled using a linear time invariant system as the convolution of the neural signals with the evoked hemodynamic response as

7 $$\begin{aligned} \varvec{Y_t}= (\varvec{x} * \varvec{h})(t) + \varvec{\varepsilon }_t, \end{aligned}$$

where $\mathbf{x}(t)$ indicates a $p \times 1$ vector of neuronal activation levels and $\mathbf{h}(t)$ is the $p\times 1$ vector containing the values assumed by the hemodynamic response function (HRF) in each ROI. In task-based fMRI data, *x*(*t*) corresponds to the stimulus function, and thus (7) coincides with the general linear model (GLM) formulation of an experimental design with *K* stimuli, first introduced by Friston et al. (1994), $\varvec{Y}_t=\sum _{k=1}^K \varvec{X}_t^{k}\circ \varvec{\beta }_{k} + \varvec{\varepsilon }_t,$, where $\circ$ represents the element-wise product of two vectors and $\varvec{\beta }_k$ is a *p*-dimensional vector of regression coefficients, representing the change in signal as a response to the *k*-th stimulus. In resting-state fMRI data, where no explicit task is being performed, the function *x*(*t*) represents latent unmeasured neural signal, to take into account the confounding effect that cardiac pulsation, respiration and the vascular architecture of the brain may induce on temporal correlations. The HRF is either assumed to take a fixed canonical shape or modeled nonparametrically as a smooth combination of basis functions. In practical settings, one can assume that the mean response signal, $(\varvec{x} * \varvec{h})(t)$, in (7) has been estimated and regressed out as a pre-processing step, so to focus on the estimation of the dynamics of the graph structures, as explained below.

In order to estimate the connectivity networks that characterize a subject under different conditions, Warnick et al. (2018) model the noise term $\varepsilon _t$ in (7) as a *p*-dimensional multivariate time-series with non-null cross-correlations. More specifically, they assume $\varepsilon _t$ as normally distributed with mean zero and variance covariance structure specified by means of a precision matrix encoding a conditional dependence structure (Lauritzen 1996b). The non-zero elements of the precision matrix correspond to edges in the connectivity network, whereas the zero elements denote conditional independence relationships between two ROIs at time *t*. To characterize possibly distinct connectivity states, i.e., network structures, within different time blocks, Warnick et al. (2018) further assume that at each time $t=1, \ldots , T$, the subject’s connectivities are described by one of $S>0$ possible states. For example, in task-based fMRI data the different states may corresponds to specific network connections activated by a stimulus, so it may be appropriate to set $S=K$. Let us introduce a collection of auxiliary latent variables $s_t \in {\mathcal {S}}\equiv \{1,\ldots , S\}$, to represent the connectivity state active at time $t=1, \ldots , T$. Then, conditionally upon $s_t$, the variance covariance structure of the *p* brain regions is described by a Gaussian graphical model by assuming

8 $$\begin{aligned} (\varvec{\varepsilon }_t | s_t=s) \sim N_p(0,\varvec{\varOmega }_s), \end{aligned}$$

where $\varvec{\varOmega }_s\in {\mathbb {R}}^p \times {\mathbb {R}}^p$ indicates a symmetric positive definite precision matrix whose zero elements encode conditional independences between the *p* components for each condition *s*, $s\in S$. Those conditional independences can be represented by the absence of edges in the underlying connectivity graphs, $G_s$, $s\in S$, which represent the brain networks. The model is completed by specifying a prior on the state-specific precision matrices $\varOmega _s$, according to the conditional dependences encoded by the underlying graphs $G_s$. For that, Warnick et al. (2018) employ the joint graphical modeling approach of Peterson et al. (2015), linking the estimation of the graph structures via a Markov random field (MRF) prior which allows, whenever appropriate, to share information across the individual brain connectivity networks in the estimation of the graph edges. Thus, the estimation of the active networks between two change points is obtained by borrowing strength across related networks over the entire time course of the experiment, also avoiding the use of post-hoc clustering algorithms for estimating shared covariance structures.

### Discussion of alternative approaches

Model frameworks based on MRF priors have two main advantages: firstly, the model learns which groups have a shared graph structure, secondly, the model exploit network similarity in the estimation of the graph for each group. These two features translate in an improved accuracy of network estimation (Peterson et al. 2015).

These approaches have been extend in several directions. For example, Shaddox et al. (2020) developed a graphical modeling framework which enables the joint inference of network structures when there is heterogeneity among both subsets of subjects (disease stage, in the motivating example) and sets of variables defined by which platform was used for measurements (gene expression and metabolite abundances, in the motivating example). The approach proposed by Shaddox et al. (2020) learns a network for each subgroup-platform combination, encourages network similarity within each platform using an MRF prior, and then links the measures of cross-group similarity across platforms.

Alternative methods for multiple graphical models, not based on MRF priors, have been proposed in the statistical literature. In the context of Gaussian DAGs, Yajima et al. (2014) propose a Bayesian method for the case of two sample groups; one group is considered the baseline group and is represented by the baseline DAG, and the DAG for the differential group is defined by a differential parameter for each possible connection. In the same context, Mitra et al. (2016) propose an alternative approach for two group structures, that allows the model to capture both network heterogeneity and to borrow strength between groups when supported by the data. A rather different approach to the Bayesian inference of multiple DAGs was proposed by Oates et al. (2016), that performed exact estimation of DAGs using integer linear programming.

Castelletti et al. (2020) develop an approach for multiple DAGs that does not rely on a fixed ordering of the nodes, and directly deals with Markov-equivalent classes. Each equivalent class is represented by an essential graph, and a novel prior on these graphs’ skeletons is used to model dependencies between groups.

In Ni et al. (2018b), they extend multiple DAGs to multiple directed *cyclic* graphs for which information is shared across multiple groups with Bayesian hierarchical formulation.

For time series data, multivariate vector autoregressive (VAR) models are used to regress current values on lagged measurements, i.e. $\mathbf{y}_t \leftarrow (\mathbf{y}_{t-1}, \ldots , \mathbf{y}_{t-s})$. These models can be represented as graphical models via a one-to-one representation between the coefficients of the VAR model and a DAG, i.e. $y_{t-s}^j\rightarrow y^i_t \iff \beta _{s,ij}\ne 0$. In Bayesian approaches, variable selection priors can be used to select the non-zero coefficients. For example, Chiang et al. (2017) employed a VAR model formulation to infer multiple brain connectivity networks based on resting-state functional MRI data measured on groups of subjects (healthy vs diseased). The variable selection approach designed by the authors allows for simultaneous inference on networks at both the subject- and group-level, while also accounting for external structural information on the brain.

In the context of multiple undirected graphs, Tan et al. (2017) consider a model based on a multiplicative prior on graph structures (Chung-Lu random graph) that links the probability of edge inclusion through logistic regression. Williams et al. (2019) propose a model for multiple graph aimed at the detection of network differences. This goal was achieved using two alternative methods for network comparison: one measured network discrepancy as the Kullback-Leibler divergence of posterior predictive distributions, whereas the second approach uses Bayes factors. Peterson et al. (2020) propose an approach which define similarity in terms of the elements of the precision matrices across groups, rather than on the binary indicators of presence of those edges; this approach is based on a novel prior on multiple dependent precision matrices.

Alternatively, approaches based on penalized likelihood that encourage either common edge selection or precision matrix similarity by penalty term on the cross-group differences were proposed by Guo et al. (2011), Zhu et al. (2014), and Cai et al. (2015). The method proposed by Danaher et al. (2014) is based on convex penalization terms that encourage similar edge values (the fused graphical lasso) or shared structure (the group graphical lasso). An underlying assumption of these methods is that all groups are related. While penalization-based methods usually scale better than their Bayesian counterpart, uncertainty in network selection is not directly assessed.

## Covariate-dependent graphs

In many applications of graphical models such as genomics and economics, covariates (say $\varvec{X}$) are often available in addition to the variables ($\varvec{Y}$) of main interest (termed response variables hereafter). For example, in cancer genomic studies, $\varvec{Y}$ represent a set of genes/proteins of which the regulatory and associative relationships are of interest and $\varvec{X}$ are clinically relevant biomarkers which could include metrics of disease severity e.g. cancer stage, subtype of cancer, or prognostic information. These biomarkers can help explain the heterogeneity among the cancer patients, that is manifested through their genomic networks. Let $\varvec{x}_l$ and $\varvec{y}_l$ denote the realizations of $\varvec{X}$ and $\varvec{Y}$ for subjects $l=1,\dots ,n$. Traditional graphical model approaches would ignore the covariates $\varvec{x}_l$ and treat $\varvec{y}_l$ as iid random variables, $\varvec{y}_l\buildrel \text{ iid }\over \sim p(\varvec{y}_l|G)$. However, the iid assumption is violated when the population is heterogeneous. To explicitly account for sampling heterogeneity, a more appropriate approach would be to introduce subject-specific graphs $G_l$ and assume $\varvec{y}_l\sim p(\varvec{y}_l|G_l)$ follows a subject-level graphical model, for each subject *l*. However, since the graph is subject-specific, without additional modeling assumptions, $G_l$ cannot be estimated with sample size one.

There are a few existing approaches that aim to solve this “sample size one” graph estimation problem. Among them, the most general framework is the graphical regression (GR) model (Ni et al. 2019). GR leverages covariates $\varvec{x}_l$ in modeling subject-level DAGs $G_l$. Because of its generality, we will first discuss the details of GR in Sect. 4.1 and then review alternative methods in Sect. 4.3, which are conceptually special cases of GR.

### Graphical regression

The main idea of GR is to formulate the inestimable subject-level parameters as functions of covariates. The functions are parameterized by population-level parameters that are shared across all subjects, thus borrowing strength and are therefore estimable. We discuss this in the context of directed graphical models (DAG) here, however, similar principles can be adapted to the undirected case as well. Specifically, GR assumes that the response variables $\varvec{y}_l$ follow a DAG model with graph $G_l$ and parameters $\varvec{\theta }_l$. Let $\varvec{y}=\{\varvec{y}_l\}_{l=1}^n$ and $\varvec{x}=\{\varvec{x}_l\}_{l=1}^n$ respectively denote the collection of $\varvec{y}_l$ and $\varvec{x}_l$ across *n* subjects. Let $pa_l(j)$ be the parent set of node *j* in graph $G_l$ and let $\varvec{y}_{l pa_l(j)}=\{y_{lk}|k\in pa_l(j)\}$ . Given the DAG $G_l$, the joint distribution admits a convenient factorization $p(\varvec{y})=\prod _{l=1}^n\prod _{j=1}^pp(y_{lj}|\varvec{y}_{l pa_l(j)},\varvec{\theta }_l)$. Assuming a linear DAG, the conditional distribution $p(y_{lj}|\varvec{y}_{l pa_l(j)},\varvec{\theta }_l)$ can be expressed as a linear regression model following Sect. 2.3,

$$\begin{aligned} y_{lj} = \sum _{k \in pa_l(j)}y_{lk}\theta _{ljk}+\epsilon _j, \end{aligned}$$

where $\theta _{ljk}$ is the strength of edge $k\rightarrow j$ in $G_l$ and $\epsilon _j\sim N(0,\sigma _j^2)$. The factorization implies all directed Markov properties encoded in $G_l$. It also indicates that $\theta _{ljk}\ne 0$ if and only if $k\rightarrow j$ and therefore learning graph $G_l$ is equivalent to finding which $\theta _{ljk}$’s are zeros or non-zeros. Again, it is clear from the regression model that the subject-level parameter $\theta _{ljk}$ cannot be estimated without further assumptions.

To address this issue, GR assumes the edge strength $\theta _{ljk}=\theta _{jk}(\varvec{x}_l)$ to be a function of covariates $\varvec{x}_l$. The function $\theta _{jk}(\cdot )$ is called *conditional independence function* (CIF) because $I\{\theta _{jk}(\varvec{x}_l)=0\}$ determines the DAG structure $G_l$ which in turn encodes the Markov properties (i.e., conditional independence relationships) of $\varvec{y}_l$ as a function of $\varvec{x}_l$. In essence, GR generalizes the (scalar) precision parameters in regular graphical models to functionals (of covariates) to model subject-specific graphs.

The specification of the functional form of $\theta _{jk}(\cdot )$ is crucial for inference of the subject-level graph $G_l$. Three properties are desired for $\theta _{jk}(\cdot )$: (i) smoothness - similar covariates should lead to similar edge strength, (ii) sparsity - the resulting graphs $G_l$ should be sparse for all *l*, and (iii) asymptotic justification - the graph (structural) recovery performance should improve as sample size increases. To equip $\theta _{jk}(\cdot )$ with these three properties, GR makes the following specific choice by decomposing $\theta _{jk}(\cdot )$ into two components,

9 $$\begin{aligned} \theta _{jk}(\varvec{x}) = f_{jk}(\varvec{x})I(|f_{jk}(\varvec{x})|>t_{jk}), \end{aligned}$$

with (i) a smooth function $f_{jk}(\cdot )$ of $\varvec{x}$ to allow for both linear and nonlinear covariate effects and (ii) a hard thresholding function with a thresholding parameter $t_{jk}$ to induce sparsity in the resulting graph structures. By construction, $\theta _{jk}(\cdot )$ is (piecewise) smooth and sparse. The asymptotic justification will be discussed after we introduced the prior distributions. GR is a fairly flexible class of models and has at least five special cases:

1. If $\varvec{x}$ is empty, then GR reduces to the case of the ordinary Gaussian DAG model (as defined in Sects. 2.3 and 2.4).
2. If $\varvec{x}$ is discrete (e.g., binary/categorical group indicator), then $\theta _{jk}(\varvec{x})$ is group-specific and GR is a multiple-DAG model (as defined in Sect. 3).
3. If $\varvec{x}$ is taken to be one of the nodes in the graphs, then GR can be interpreted as a context-specific DAG (Geiger and Heckerman 1996).
4. If the distribution of $\theta _{jk}(\varvec{x})$ is absolutely continuous with respect to Lebesgue measure, then GR is a conditional DAG model in which the strength of the graph varies continuously with the covariates but the structure is constant.
5. If $\varvec{x}$ is univariate time points, then GR can be used for modeling time-varying DAGs.

A variety of approaches (parametric or non-parametric) are available to model the smooth function $f_{jk}(\cdot )$ in a flexible manner. One attractive parameterization that is tractable both interpretationally and computationally is using penalized splines (p-splines) with orthogonal basis expansions. Specifically, suppose $\varvec{x}=(x_1,\dots ,x_Q)$ is *Q*-dimensional. They first expand $f_{jk}(\varvec{x})$ using additive cubic b-splines $f_{jk}(\varvec{x})=\sum _{q=1}^Q f_{jkq}(x_q)$ with $f_{jkq}(x_q)=\widetilde{\varvec{x}}_q^T\varvec{\beta }_{jkq}$ where $\widetilde{\varvec{x}}_q$ are the b-spline bases of $x_q$ and $\varvec{\beta }_{jkq}$ are the spline coefficients. A relatively large number *B* of bases are chosen so that local features can be captured and a roughness penalty is imposed to prevent overly complex curve fitting. In the Bayesian paradigm, the penalty is implemented through a Gaussian random walk prior on the spline coefficients, $\varvec{\beta }_{jkq}\sim N(0,s\varvec{K}^-)$ where $\varvec{K}$ is obtained from the second order differences of adjacent spline coefficients and the superscript “−” denotes psuedo-inverse. In order to differentiate linear covariate effects from nonlinear effects, the b-spline bases $\widetilde{\varvec{x}}_q$ are orthogonalized into a “purely” nonlinear bases $\widetilde{\varvec{x}}_q^{\star }$ that is orthogonal to the linear term $x_q$. As a result, $f_{jkq}(x_q)$ is decomposed as $f_{jkq}(x_q)=f_{jkq}^{\star }(x_q)+f_{jkq}^0(x_q)=\widetilde{\varvec{x}}_q^{\star T}\varvec{\beta }_{jkq}^\star +x_q\beta _{jkq}$. To select important covariates, spike-and-slab priors are imposed on $\varvec{\beta }_{jkq}^\star$ and $\beta _{jkq}$. Let $v_0$ be a fixed small number. The linear effect $\beta _{jkq}$ follows,

$$\begin{aligned}&\beta _{jkq}\sim \gamma _{jkq} N(0,v_{jkq})+(1-\gamma _{jkq})N(0,v_0v_{jkq}),\\&\quad \gamma _{jkq}\sim \text{ Beta-Bernoulli }(a_\gamma ,b_\gamma ), ~~v_{jkq}\sim IG(a_v,b_v), \end{aligned}$$

where the binary indicator $\gamma _{jkq}$ indicates the significance of linear effect of covariate $x_q$ on edge $j\leftarrow k$. For the nonlinear effects, a parameter-expansion technique is used, $\varvec{\beta }_{jkq}^\star =\eta _{jkq}^\star \varvec{\xi }_{jkq}$ where $\eta _{jkq}^\star$ is a scalar and has the same prior as $\beta _{jkq}$,

$$\begin{aligned}&\eta _{jkq}^\star \sim \gamma _{jkq}^\star N(0,v_{jkq}^\star )+(1-\gamma _{jkq}^\star )N(0,v_0v_{jkq}^\star ), \\&\quad \gamma _{jkq}^\star \sim \text{ Beta-Bernoulli }(a_\gamma ,b_\gamma ), ~~v_{jkq}^\star \sim IG(a_v,b_v), \end{aligned}$$

and

$$\begin{aligned} \varvec{\xi }_{jkq}\sim N(\varvec{m}_{jkq},\varvec{I}_q),~~m_{jkq b}\sim 0.5\delta _1+0.5\delta _{-1}, \end{aligned}$$

where $\varvec{m}_{jkq}=(m_{jkqb})_{b=1}^B$. Similarly to linear effects, the binary indicator $\gamma _{jkq}^\star$ indicates the significance of nonlinear effect of covariate $x_q$ on edge $j\leftarrow k$ (through the magnitude of $\eta _{jkq}^\star$). The vector $\varvec{\xi }_{jkq}$ distributes $\eta _{jkq}^\star$ across the entries of $\varvec{\beta }_{jkq}^*$. The model is completed by assigning a conjugate inverse-gamma $\sigma _j^2\sim IG(a_\sigma ,b_\sigma )$ and a standard MCMC algorithm is used to sample all the model parameters from the posterior distribution.

While the spike-and-slab priors induce sparsity in the covariate effects, they do not necessarily give rise to a sparse DAG $G_l$. The hard thresholding function in (9) is crucial in introducing extra sparsity in DAGs. The thresholding parameter $t_{jk}$ controls the sparsity and can be interpreted as the minimum effect size of the CIF. In principle, $t_{jk}$ can be fixed or assigned a prior distribution. The latter is preferred because (i) the minimum effect size is rarely known in practice, and (ii) a wide range of priors on $t_{jk}$ induce a non-local prior on $\theta _{jk}$ which in turn leads to selection consistency under several regularity conditions – see (Ni et al. 2019) for further details.

*Graph prediction* Another novel feature of GR is that it can be used to predict graph structure for new data points. It is achieved through the posterior predictive distribution of the CIF $\theta _{jk}(\varvec{x}^{new})$ which can be approximated by MCMC samples (indexed by superscript “(s)”),

10 $$\begin{aligned} Pr\{\theta _{jk}(\varvec{x}^{new})\ne 0|\varvec{y},\varvec{x}\}\approx \frac{1}{S}\sum _{s=1}^SI\{\theta _{jk}^{(s)}(\varvec{x}^{new})\ne 0\}. \end{aligned}$$

Notice that equation (10) does not depend on $\varvec{y}^{new}$, and therefore structure prediction requires new covariates $\varvec{x}^{new}$ only. In practice, this is a desirable property. For example, one can predict the gene network for new patients without sequencing the whole genome; the measurement of external covariates (e.g, prognostic factors) will suffice.

### Application of graphical regression to multiple myeloma genomics data

To illustrate the utility and versatility of GR we use the same dataset in Sect. 3.2 with the goal of constructing a subject-specific graph by incorporating prognostic factors S$\beta _2$M and serum albumin. We run two independent Markov chains, each for 500,000 iterations ( 47 hours), discard the first 50% as burn-in, and thin the chain by taking every 25th sample.

The inferred network is shown in Fig. 2d. We find (i) 38 positive constant edges (solid lines with arrowheads), (ii) 20 negative constant edges (solid lines with flat heads), (iii) 2 edges linearly varied with covariates (dashed lines), and (iv) 9 edges nonlinearly varied with covariates (dotted lines). The width of the solid lines (constant edges) is proportional to its posterior probability. Some regulatory relationships are consistent with those reported in the existing biological literature. For example, NRAS/HRAS activating MAP2K2 is part of the well-known MAPK cascade, which participates in the regulation of fundamental cellular functions, including proliferation, survival and differentiation. Mutated regulation is a necessary step in the development of many cancers (Roberts and Der 2007). We also observe that IL6R activates PIK3R1, which together with its induced PI3K/AKT pathway plays a key role in protection against apoptosis and the proliferation of multiple myeloma cells (Hideshima et al. 2001). Moreover, we find two driver/hub genes, FLT4 and MAP2K3 with degrees 9 and 8, both of which play important roles in multiple myeloma. FLT4, also known as VEGFR3, is responsible for angiogenesis for multiple myeloma (Kumar et al. 2003) and MAP2K3 contributes to the development of multiple myeloma through MAPK cascades (Leow et al. 2013).

**Fig. 3:**
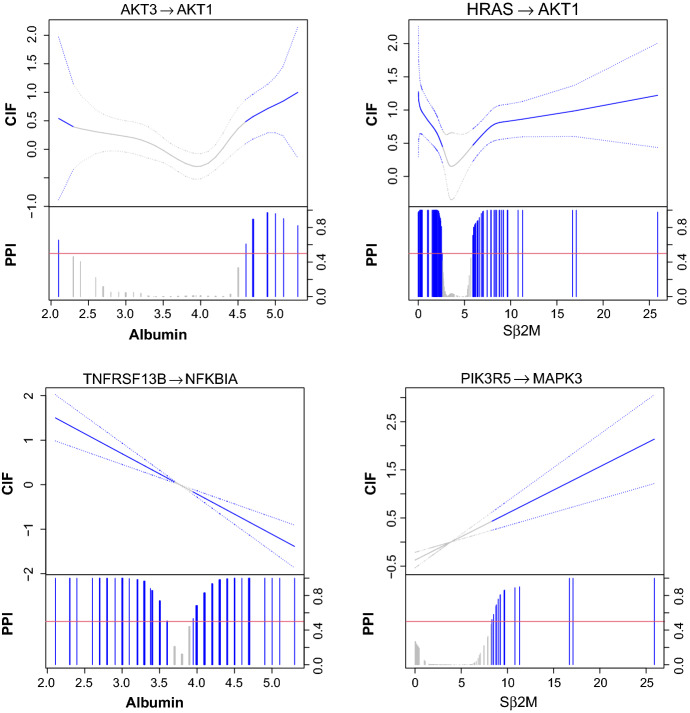
Nonlinearly (top) and linearly (bottom) varying effects for the multiple myeloma dataset analyzed by the graphical regression model. For each plot, the estimated conditional independence functions (solid) with 95% credible bands (dotted) are shown in the top portion and marginal posterior inclusion probabilities are shown in the bottom portion. Red horizontal line is the 0.5 probability cutoff. Blue (grey) lines and curves indicate (in)significant coefficients

*Varying gene regulations.* A unique output of graphical regression analysis compared to multiple graphical model is the inference of continuously varying gene regulation as functions of external covariates. In Fig. 3, we present two nonlinearly varying and two linearly varying effects. There is an interesting nonlinear relationship between HRAS and AKT1 as a function of S$\beta _2$M. Prior work indicates that RAS may activate the AKT pathway in multiple myeloma (Hu et al. 2003). We find that HRAS upregulates AKT1 when S$\beta _2$M<2.64 or S$\beta _2$M>5.70 but the regulatory relationship becomes insignificant when 2.64<S$\beta _2$M$<5.70$ (i.e., primarily the stage II multiple myeloma patients). The linear relationship between TNFRSF13B and NFKBIA is also interesting. Many multiple myeloma studies (Silke and Brink 2010) have revealed the importance of NF-$\kappa$B activation, the inhibitor of which, NFKBIA, is degraded by TNFRSF (TNFRSF13B is a member of TNFRSF). We find that the sign of the regulation switches at around 3.5 g/dL of serum albumin that distinguishes between stages I and II. As expected, when serum albumin concentrations become higher, which suggests more advanced multiple myeloma, the inactivation becomes stronger.

### Discussion of alternative covariate-dependent graphs

We now discuss several alternative approaches (Liu et al. 2010; Kolar et al. 2010a; Zhou et al. 2010; Kolar et al. 2010b; Cheng et al. 2014) that also account for heterogeneity by utilizing the covariates $\varvec{X}$.

In Liu et al. (2010), they proposed to partition the covariate space $\varvec{X}\in {\mathcal {X}}$ into disjoint subspaces ${\mathcal {X}}=\cup _{k=1}^K {\mathcal {X}}_k$ using decision tree and then fit a Gaussian graphical model independently to each subspace, $\prod _{k=1}^K\prod _{i:\varvec{x}_i\in {\mathcal {X}}_k}p(\varvec{y}_i|G_k)$ where $G_k$ denotes an undirected graph specific to subspace *k*. Compared to the graphical regression framework, this approach may lead to very different graphs for similar covariates due to the independent graph estimation.

Kolar et al. (2010a) proposed a penalized kernel smoothing approach for conditional Gaussian graphical models in which the precision matrix varies with the continuous covariates. Cheng et al. (2014) developed a conditional Ising model for binary data where the dependencies are linear functions of additional covariates. Although these two methods allow the edge strength to vary with the covariates, their graph structures stay constant. Zhou et al. (2010) and Kolar et al. (2010b) proposed time-varying undirected graph algorithms for time series data. The graph structure is allowed to change over time by borrowing strength from “neighboring” time points via kernel smoothing. The graph estimation problem is essentially broken down to separate estimation for each time point. Because of the reliance on kernel smoothing, extension to a large number of covariates requires careful redesign of the models to mitigate the curse of dimensionality.

Additionally, there are graphical models that incorporate covariates not necessarily for the purpose of accounting for heterogeneity. In Ni et al. (2018a), they exploit the prior biological knowledge and covariates (DNA methylation and DNA copy number) to identify cyclic causal gene regulatory relationships. Note that covariate-dependent graphs differ fundamentally from chain graphs; the latter type is discussed in the next section.

## Other complex networks

In this section we discuss a range of techniques for the analysis of networks for scenarios that go beyond what discussed in the previous sections. More specifically, we focus on robust graphical models, array/matrix-variate graphical models, and chain graphs. In the last part of this section, we also discuss how to integrate graphical and regression models.

### Robust graphical models

Some robust graphical models exist in the literature for the analysis of data that show departure from Gaussianity due to the presence of outliers or spikes in the data that can lead to inaccurate estimation of the graphs. For example, Pitt et al. (2006) used copula models and Bhadra et al. (2018) used Gaussian scale mixtures. Here, we briefly describe the approach of Finegold and Drton (2011, 2014), who employ positive latent contamination parameters (divisors) to regulate the departure of the data from Gaussianity. The approach assumes multivariate-t distributions for the data. Let $\varvec{y}$ follow a classical multivariate-t distribution $t_{p}(\varvec{\mu }, \varvec{\varOmega }^{-1}, \nu )$ with $\nu$ degrees of freedom, mean $\varvec{\mu }$, and a $p\times p$ matrix $\varvec{\varOmega }^{-1}$. This distribution is equivalent to

11 $$\begin{aligned} \begin{aligned}&(\varvec{y}_l|\tau _l) \sim N_p(\varvec{0},\varvec{\varOmega }^{-1}/\tau _l),\\&\quad (\tau _l| \nu ) \overset{iid}{\sim } \text {Gamma}(\nu /2,\nu /2), \quad l = 1,...,n, \end{aligned} \end{aligned}$$

with scaling parameters $\tau _l$ that downweight the extreme values in the data. In the classical-t graphical model of Finegold and Drton (2011), a graph ${\mathcal {G}}$ is determined by the zeros in $\varvec{\varOmega }$, similarly to the Gaussian case. A disadvantage of the classical-t distribution model is that it reweights all *p* dimensions of $\varvec{y}_l$ by the same scale parameter. In Finegold and Drton (2014) the authors address this problem by employing subject-specific vectors $\varvec{\tau }_l = (\tau _{l1}, \tau _{l2},..., \tau _{lp} )$ that scale each of the *p* dimension of $\varvec{y}_l$ separately. In order to increase model flexibility and avoid over-parameterization, Dirichlet Process (DP) priors are imposed on $\varvec{\tau }_l$ to enforce clustering when suggested by the data. This results in the Dirichlet-t graphical model

12 $$\begin{aligned} \begin{aligned}&(\varvec{y}_l|\varvec{\tau }_l) \sim N_p(\varvec{0}, \text {diag}( 1/ \sqrt{\varvec{\tau }_l}) \cdot \varvec{\varOmega }^{-1} \cdot \text {diag}( 1/ \sqrt{\varvec{\tau }_l})),\\&\quad \tau _{lj} \overset{iid}{\sim } P_l, \quad j=1,...,p,\\&\quad P_l \overset{iid}{\sim } DP(\alpha , P_0),\quad l=1,...,n,\\&\quad P_0 = \text {Gamma}(\nu /2,\nu /2),\\&\quad \alpha \sim \text {Gamma}(a_\alpha , b_\alpha ). \end{aligned} \end{aligned}$$

This model formulation, however, does not allow the exchange of information among the vectors of observed data, since independent Dirichlet process priors are used for each of the *n* samples. Cremaschi et al. (2019) improve on this model by using a hierarchical construction based on a more flexible class of nonparametric prior distributions, known as normalized completely random measures (NormCRMs), first introduced by Regazzini et al. (2003). Furthermore, Bhadra et al. (2018) allow extensions to mixtures of continuous and discrete-valued (binary or ordinal) nodes through a latent variable framework for inferring conditional independence structures.

### Matrix and tensor graphical models

There are many other settings where random variables/responses are measured along multiple axes or dimensions (e.g. space, time). The resulting observed data can be then construed as a matrix or a tensor. For example, consider an experiment in which a set of cell lines, the statistical units, is exposed to a set of *K* treatments; the expression of *p* genes is measured from all cell lines. This is the typical case of a multi-dimensional structure that encodes dependencies among observed variables that are not interchangeable across dimensions and require new methodological developments.

Ni et al. (2017) developed a multi-dimensional graphical model for tensor data $\varvec{Y}\in {\mathbb {R}}^{p_1\times p_2\times \cdots \times p_K}$ which allows for simultaneous construction of graphs along all dimensions. The graphs can be directed, undirected, or arbitrary combinations of the two. To introduce the model, let us first consider a centered array-variate normal distribution, $\varvec{Y}\sim N(\varvec{0},\varOmega _1^{-1},\dots ,\varOmega _K^{-1})$ where $\varOmega _k$ is the precision matrix of dimension $k=1,\dots ,K$. Let $\varvec{Z}=vec(\varvec{Y})$ be the vector obtained by stacking the elements of $\varvec{Y}$ in the order of its dimensions. The array-variate normal distribution of $\varvec{Y}$ is equivalent to a multivariate normal of $\varvec{Z}$ with a separable precision matrix with respect to Kronecker product, $\varvec{Z}\sim N(\varvec{0},\varOmega _K^{-1}\otimes \cdots \otimes \varOmega _1^{-1})$. Then they define an array-variate DAG model by a tensor structural equation model,

13 $$\begin{aligned} (B_K\otimes \cdots \otimes B_1)\varvec{Y}=\varvec{E}~~\text{ with }~~\varvec{E}\sim N(\varvec{0},T_K\otimes \cdots \otimes T_1), \end{aligned}$$

where $B_k$ is an upper triangular matrix with unit diagonal entries and $T_k$ is a diagonal matrix with positive entries. It is not difficult to see that $\varOmega _k=B_k^TT_k^{-1}B_k$ which is the modified Cholesky decomposition of $\varOmega _k$. To ensure identifiability the last element of $T_k$ is fixed to 1 for all *k*. Importantly, the sparsity of $B_k$ corresponds to the graph structure $G_k$ of dimension *k*. More precisely, $B_{kij}\ne 0$ if and only if $i \rightarrow j$ in graph $G_k$. The array-variate DAG model in (13) encodes the conditional independence relationships among the variables along each dimension which can be read off from graph $G_k$ using the notion of d-separation.

Model (13) can be also used for constructing undirected (decomposable) graphs due to the equivalence between decomposable graphs and *perfect* DAGs. A set *R* denote of pairs of indices is said to be reducible if $\forall (i, j) \in R$ with $i < j$, either $(h, i) \in R$ or $(h, j) \in R$, $\forall h = 1,\dots , i - 1$. The null set *R* with respect to a matrix *M* is defined as $R = \{(i, j)|M_{i j} = 0\}$. Then an undirected graph $G_k$ is decomposable if and only if there exists an ordering of $\varOmega _k$ such that $B_k$ has the same reducible null set as $\varOmega _k=B_k^TT_k^{-1}B_k$. Since (13) also implies $\varOmega _k=B_k^TT_k^{-1}B_k$, the array-variate decomposable Gaussian graphical models can also be represented by (13) with a proper chosen ordering of $G_k$ which can be obtained by maximum cardinality search algorithm. Because (13) provides a unified framework for modeling both directed and undirected graphs through directed graphs, no additional treatment is required for a hybrid array-variate graphs where some of $G_k$’s are directed and others are undirected.

In order to make posterior inference of the graph structures, spike-and-slab priors are used, $B_{kij}\sim \gamma _{kij}N(0,\tau _kT_k)+(1-\gamma _{kij})\delta _0$ with $\gamma _{kij}\sim \text{ beta-Bernoulli }(a_\rho ,b_\rho )$. The binary parameter $\gamma _{kij}$ indicates whether $i \rightarrow j$ or $i - j$ is present in graph $G_k$. The model is completed with independent inverse-gamma priors on the entries of $T_k$. Partially collapsed Gibbs sampler (Van Dyk and Park 2008) is adopted to efficiently explore the posterior graph space.

### Chain graphical models

Chain graphs are another popular type of graphs; variables are grouped in chain components that follow a given ordering. Within a chain component, variables are connected by undirected edges, and arrows connect variables in a parent component to variables in a child component. In recent years, methods for the analysis of high-dimensional chain graphs have been proposed, many of which focused on two-component graphs. For example, in Rothman et al. (2010); Yin and Li (2011); Bhadra and Mallick (2013), they propose conditional Gaussian graphical models that are in essence multivariate linear regression models with the error terms following an iid undirected Gaussian graphical model. However, note that while the graph estimation is conditional on the covariates, they only enter the model via the mean structure, a fundamental difference with respect to the models presented in Sect. 4.1. As a consequence, the graph topology and the precision matrix stay the same across observations. Motivated by the analysis of multi-platform genomics data, Ha et al. (2020) proposed a Bayesian approach for chain graph selection based on node-wise likelihoods that converts the chain graph into a more tractable multiple regression model, accounting for both with and between chain component dependencies. In a chain graph, the probability distribution of the observed random variables $\varvec{Y}$ can be factorized as $P(\varvec{Y}) = \prod _{\tau \in \mathcal {T}} P(\varvec{Y}_{\tau }|\varvec{Y}_{\texttt {pa}_\tau })$, where $\tau$ represent chain components belonging to the ordered partitioning $\mathcal {T}$ (Lauritzen 1996a). Under the normality assumption $Y \sim N(\varvec{0},\varvec{\varOmega }^{-1})$, a chain graph $G=(V,E)$, and the AMP Markov properties (Andersson et al. 2001), we have

14 $$\begin{aligned} \varvec{Y} = \mathbf {B}\varvec{Y} + \varvec{\epsilon } \text {, } \varvec{\epsilon }\sim N(\varvec{0},\varvec{\mathcal {K}}^{-1}), \end{aligned}$$

where $\mathbf {B}=(b_{vu})$ is a $p\times p$ matrix for which the zero pattern encodes the directed edges between chain components, and the precision matrix of $\varvec{\mathcal {K}}$ is a matrix for which the nonzero off-diagonal elements represent the undirected edges within a chain component after taking into account the effects from the directed edges.

Ha et al. (2020) derived a node-wise likelihood that, for a given node *v*, can be written as

$$\begin{aligned} Y_v = \varvec{Y}_{\mathcal {P}_v}^\mathrm {T}\mathbf {b}_v + \varvec{Y}_{\mathcal {C}_v}^\mathrm {T}\varvec{\alpha }_v -\varvec{Y}_{\mathcal {P}_v}^\mathrm {T}\mathbf {B}^\mathrm {T}_{\mathcal {C}_v,\mathcal {P}_v} \varvec{\alpha }_v + e_v, \end{aligned}$$

where $\varvec{\alpha }_v = -\varvec{\mathcal {K}}_{\mathcal {C}_v,v}/\kappa _{vv}$, $\mathcal {C}_v$ and $\mathcal {P}_v$ are defined by the set of all other vertices in the same layer as *v* and all the preceding vertices, $C_{t(v)-1}$, respectively, and $e_v\sim N(0,1/\kappa _{vv})$ is independent of all other random variables; see Ha et al. (2020) for technical details. Within this framework, the undirected and directed edges of the chain graph can be selected using zero restrictions on the regression parameters, $\mathbf {B}$ and $\varvec{\alpha }$. Standard selection priors, such as spike-and-slab, and companion algorithm can be implemented for inference and model selection. This approach results in a computationally efficient algorithm that can be used for the analysis of large graphs.

### Integrative analysis of graphical and regression models

Regression models are often used when it is required to predict a response variable, either univariate or multivariate, given a potentially large set of covariates. Regression models with fixed covariates are typically used; this is equivalent to estimate the distribution of the response variable conditionally upon the observed values of the covariates. In many scientific areas, such as genomics and imaging, models that account for the dependence structure among the covariates have been shown to provide a deeper understanding of the data generating mechanisms as well as to have improved prediction performances. The dependence structure of the covariates can be learned from the data and represented by a graphical model. In the context of cancer integrative genomics, Chekouo et al. (2015) developed a model for the analysis of time to event responses that uses gene and microRNA expression as predictors; the dependence structure between gene and microRNA is represented by a DAG, inferred from the data, and this DAG is used to drive the selection of covariates relevant for the prediction of the response variable. Interestingly, covariates connected in the DAG are more likely to be selected.

Peterson et al. (2016) proposed a general Bayesian framework for the selection of covariates that are connected within a undirected graph; the graph itself is estimated from the data. The flexibility of this model is particularly useful in genomics applications, since the estimated network among the covariates can encourage the joint selection of functionally related genes (or proteins).

A similar approach can be very effective for the analysis of imaging genetics data. Chekouo et al. (2016) investigated genetic variants and imaging biomarkers that can predict a given clinical condition, such as schizophrenia. The proposed predictive model discriminates between subjects affected by the disease and healthy controls based on a subset of the imaging and genetic markers accounting for the dependence structure between these two sets of covariates. In this case the model learns and accounts for both directed and undirected associations. Accounting for the dependence structure of the covariates results in better predictions of the disease status.

## Discussion

The availability of complex-structured data from modern biomedical technologies such as genomic and neuroimaging data, has spawned many analytical frameworks that go beyond the traditional graphical modeling approaches – to better understand and characterize the dependency structures encoded in these rich datasets. In this article, we have reviewed some state-of-the-art Bayesian approaches for a variety of inferential tasks: analysis of multiple networks, network regression with covariates and other recent graphical model methods that are suited for non-standard settings. Specifically, we focused on scenarios where the number of observed units/subjects is smaller than the number of observed random variables, and for which a single network is not representative of the (global) dependency structures of the targeted population.

Inference for the discussed methods is performed via MCMC algorithms. These algorithms are used to calculate the joint posterior distribution of all parameters, a key quantity to quantify uncertainty associated to graph selection. Usually these algorithms do not scale as well as optimization approaches based on penalized likelihood; the maximum graph size that can be analyzed depends on many factors, including type of graph, statistical model and the specific dataset on hand. In the context of multiple graphs models, alternative computational strategies have been developed and relevant instances include the EM algorithm proposed by Li et al. (2020), that results in a point estimate of the graphs and can scale better to lager dimensions, and a sequential Monte Carlo (SMC) algorithm proposed by Tan et al. (2017), that has similar computational performances than its MCMC counterparts.

We have focused our article on the key methodological aspects, modeling assumptions and ensuing advantages of these approaches. We also illustrate the practical utility of some of these methods using examples in cancer genomics and neuroimaging. The companion software of the methods discussed in this review paper is available at the authors’ website or in publicly accessible repositories (links are provided in Sect. 1). Our hope is that these methods will engender future investigators in this exciting area.

Admittedly, there are several other issues and areas that we have not covered in this review. While these models are rich and flexible, we also acknowledge their limitations, including computational complexity of MCMC-based sampling algorithms and the need to specify prior distributions and hyperparameters; although the latter may be advantageous in some settings e.g. where *a priori* biological information needs to be incorporated. Finally, our focus in this article is on probabilistic graphical models, where networks reconstruction is the key objective, as opposed to inference on observed network data (Hoff et al. 2002).

## Footnotes

1 https://github.com/elinshaddox/MultiplePlatformBayesianNetworks.

2 https://www.stat.tamu.edu/~yni/files/GR_code.zip.

## References

[CR1] Altomare D, Consonni G, La Rocca L (2013). Objective bayesian search of gaussian directed acyclic graphical models for ordered variables with non-local priors. Biometrics.

[CR2] Andersson SA, Madigan D, Perlman MD (1997). A characterization of Markov equivalence classes for acyclic digraphs. The Ann Stat.

[CR3] Andersson SA, Madigan D, Perlman MD (2001). Alternative markov properties for chain graphs. Scan J Stat.

[CR4] Atay-Kayis A, Massam H (2005). The marginal likelihood for decomposable and non-decomposable graphical gaussian models. Biometrka.

[CR5] Banerjee O, El Ghaoui L, d’Aspremont A (2008). Model selection through sparse maximum likelihood estimation for multivariate gaussian or binary data. The J Mach Learn Res.

[CR6] Bhadra A, Mallick BK (2013). Joint high-dimensional Bayesian variable and covariance selection with an application to eQTL analysis. Biometrics.

[CR7] Bhadra A, Rao A, Baladandayuthapani V (2018). Inferring network structure in non-normal and mixed discrete-continuous genomic data. Biometrics.

[CR8] Boyd KD, Davies FE, Morgan GJ (2011) Novel drugs in myeloma: harnessing tumour biology to treat myeloma. In: Multiple Myeloma, Springer, pp 151–187 10.1007/978-3-540-85772-3_8 21509685

[CR9] Cai T, Li H, Liu W, Xie J (2015). Joint estimation of multiple high-dimensional precision matrices. Stat Sinica.

[CR10] Carvalho C, Polson N, Scott J (2010). The horseshoe estimator for sparse signals. Biometrika.

[CR11] Carvalho CM, Scott JG (2009). Objective Bayesian model selection in Gaussian graphical models. Biometrika.

[CR12] Castelletti F, Consonni G, Della Vedova M, Peluso S (2018). Learning Markov equivalence classes of directed acyclic graphs: an objective Bayes approach. Bayesian Anal.

[CR13] Castelletti F, La Rocca L, Peluso S, Stingo F, Consonni G (2020). Bayesian learning of multiple directed networks from observational data. Stat Med.

[CR14] Chapman MA, Lawrence MS, Keats JJ, Cibulskis K, Sougnez C, Schinzel AC, Harview CL, Brunet JP, Ahmann GJ, Adli M (2011). Initial genome sequencing and analysis of multiple myeloma. Nature.

[CR15] Chekouo T, Stingo F, Doecke J, Do KA (2015). Mirna-target gene regulatory networks: a bayesian integrative approach to biomarker selection with application to kidney cancer. Biometrics.

[CR16] Chekouo T, Stingo F, Guindani M, Do KA (2016). A bayesian predictive model for imaging genetics with application to schizophrenia. Ann Appl Stat.

[CR17] Cheng J, Levina E, Wang P, Zhu J (2014). A sparse ising model with covariates. Biometrics.

[CR18] Chiang S, Guindani M, Yeh HJ, Haneef Z, Stern JM, Vannucci M (2017). Bayesian vector autoregressive model for multi-subject effective connectivity inference using multi-modal neuroimaging data. Human Brain Map.

[CR19] Chickering DM (2002). Learning equivalence classes of Bayesian-network structures. J Mach Learn Res.

[CR20] Clyde M, George E (2004). Model uncertainty. Stat Sci.

[CR21] Cremaschi A, Argiento R, Shoemaker K, Peterson C, Vannucci M (2019). Hierarchical normalized completely random measures for robust graphical modeling. Bayesian Anal.

[CR22] Cribben I, Haraldsdottir R, Atlas L, Wager TD, Lindquist MA (2012). Dynamic connectivity regression: determining state-related changes in brain connectivity. NeuroImage.

[CR23] Danaher P, Wang P, Witten D (2014). The joint graphical lasso for inverse covariance estimation across multiple classes. J Royal Stat Soc Series B.

[CR24] Dobra A, Hans C, Jones B, Nevins JR, Yao G, West M (2004). Sparse graphical models for exploring gene expression data. J Multivar Anal.

[CR25] Dobra A, Lenkoski A, Rodriguez A (2011) Bayesian inference for general gaussian graphical models with application to multivariate lattice data. J Am Stat Assoc 106(496) 10.1198/jasa.2011.tm10465 PMC4767185 26924867

[CR26] Finegold M, Drton M (2011) Robust graphical modeling of gene networks using classical and alternative $$t$$-distributions. The Ann Appl Stat. pp 1057–1080

[CR27] Finegold M, Drton M (2014). Robust bayesian graphical modeling using dirichlet $$t$$-distributions. Bayesian Anal.

[CR28] Friedman J, Hastie T, Tibshirani R (2008). Sparse inverse covariance estimation with the graphical lasso. Biostatistics.

[CR29] Friedman N (2004). Inferring cellular networks using probabilistic graphical models. Sci Signal.

[CR30] Friedman N, Linial M, Nachman I, Pe’er D (2000). Using bayesian networks to analyze expression data. J Comput Biol.

[CR31] Friston KJ, Jezzard P, Turner R (1994). Analysis of functional MRI time-series. Human Brain Map.

[CR32] Geiger D, Heckerman D (1996). Knowledge representation and inference in similarity networks and bayesian multinets. Artif Intell.

[CR33] Geiger D, Heckerman D (2002). Parameter priors for directed acyclic graphical models and the characterization of several probability distributions. The Ann Stat.

[CR34] George E, McCulloch R (1993). Variable selection via Gibbs sampling. J Am Statist Assoc.

[CR35] Greipp PR, San Miguel J, Durie BG, Crowley JJ, Barlogie B, Bladé J, Boccadoro M, Child JA, Avet-Loiseau H, Kyle RA (2005). International staging system for multiple myeloma. J Clin Oncol.

[CR36] Griffin JE, Brown PJ (2010). Inference with normal-gamma prior distributions in regression problems. Bayesian Anal.

[CR37] Guo J, Levina E, Michailidis G, Zhu J (2011). Joint estimation of multiple graphical models. Biometrika.

[CR38] Ha MJ, Stingo FC, Baladandayuthapani V (2020) Bayesian structure learning in multi-layered genomic networks. J Am Stat Assoc **(forthcoming)** 10.1080/01621459.2020.1775611 PMC8259335 34239216

[CR39] Hanahan D, Weinberg R (2011). Hallmarks of cancer: the next generation. Cell.

[CR40] Hideshima T, Nakamura N, Chauhan D, Anderson KC (2001). Biologic sequelae of interleukin-6 induced pi3-k/akt signaling in multiple myeloma. Oncogene.

[CR41] Hoff PD, Raftery AE, Handcock MS (2002). Latent space approaches to social network analysis. J Am Stat Assoc.

[CR42] Hu L, Shi Y, Hsu Jh, Gera J, Van Ness B, Lichtenstein A (2003). Downstream effectors of oncogenic ras in multiple myeloma cells. Blood.

[CR43] Iyengar R, Altman R, Troyanskya O, FitzGerald G (2015). Personalization in practice. Science.

[CR44] Jones B, Carvalho C, Dobra A, amd C Carter CH, West M, (2005) Experiments in stochastic computation for high-dimensional graphical models. Stat Sci 20(4):388–400

[CR45] Kolar M, Parikh AP, Xing EP (2010a) On sparse nonparametric conditional covariance selection. In: Proceedings of the 27th international conference on machine learning (ICML-10), pp 559–566

[CR46] Kolar M, Song L, Ahmed A, Xing EP (2010b) Estimating time-varying networks. The Ann Appl Stat. pp 94–123

[CR47] Kumar S, Witzig T, Timm M, Haug J, Wellik L, Fonseca R, Greipp P, Rajkumar S (2003). Expression of vegf and its receptors by myeloma cells. Leukemia.

[CR48] Kundu S, Baladandayuthapani V, Mallick B (2013) Bayes regularized graphical model estimation in high dimensions. arXiv preprint arXiv:13083915

[CR49] Lauritzen S (1996). Graphical models.

[CR50] Lauritzen SL (1996b) Graphical Models. Oxford University Press

[CR51] Leow CCY, Gerondakis S, Spencer A (2013) Mek inhibitors as a chemotherapeutic intervention in multiple myeloma. Blood Cancer J 3(3) 10.1038/bcj.2013.1 PMC3615214 23524590

[CR52] Li Z, McComick T, Clark S (2020). Using Bayesian latent Gaussian graphical models to infer symptom associations in verbal autopsies. Bayesian Anal.

[CR53] Liu H, Chen X, Wasserman L, Lafferty JD (2010) Graph-valued regression. In: Lafferty JD, Williams CKI, Shawe-Taylor J, Zemel RS, Culotta A (eds) Advances in Neural Information Processing Systems 23, Curran Associates, Inc., pp 1423–1431, http://papers.nips.cc/paper/3916-graph-valued-regression.pdf

[CR54] Meinshausen N, Bühlmann P (2006) High-dimensional graphs and variable selection with the lasso. The Ann Stat pp 1436–1462

[CR55] Mitra R, Müller P, Ji Y (2016). Bayesian graphical models for differential pathways. Bayesian Anal.

[CR56] Mohammadi A, Wit E (2015). Bayesian structure learning in sparse gaussian graphical models. Bayesian Anal.

[CR57] Mohammadi A, Wit E (2019). Bdgraph: an r package for Bayesian structure learning in graphical models. J Stat Softw.

[CR58] Møller J, Pettitt A, Reeves R, Berthelsen K (2006). An efficient markov chain monte carlo method for distributions with intractable normalising constants. Biometrika.

[CR59] Mukherjee S, Speed T (2008). Network inference using informative priors. PNAS.

[CR60] Ni Y, Stingo FC, Baladandayuthapani V (2015). Bayesian nonlinear model selection for gene regulatory networks. Biometrics.

[CR61] Ni Y, Stingo FC, Baladandayuthapani V (2017). Sparse multi-dimensional graphical models: a unified bayesian framework. J Am Stat Assoc.

[CR62] Ni Y, Ji Y, Müller P (2018). Reciprocal graphical models for integrative gene regulatory network analysis. Bayesian Anal.

[CR63] Ni Y, Müller P, Zhu Y, Ji Y (2018). Heterogeneous reciprocal graphical models. Biometrics.

[CR64] Ni Y, Stingo FC, Baladandayuthapani V (2019). Bayesian graphical regression. J Am Stat Assoc.

[CR65] Oates C, Smith J, Mukherjee S, Cussens J (2016). Exact estimation of multiple directed acyclic graphs. Stat Comput.

[CR66] Peterson C, Osborne N, Stingo F, Bourgeat P, Doecke J, Vannucci M (2020) Bayesian modeling of multiple structural connectivity networks during the progression of alzheimer’s disease. Biometrics 10.1111/biom.13235 PMC8906798 32026459

[CR67] Peterson CB, Stingo F, Vannucci M (2015). Bayesian inference of multiple Gaussian graphical models. J Am Stat Assoc.

[CR68] Peterson CB, Stingo F, Vannucci M (2016). Joint Bayesian variable and graph selection for regression models with network-structured predictors. Stat Med.

[CR69] Pierson E, Consortium G, Koller D, Battle A, Mostafavi S (2015) Sharing and specificity of co-expression networks across 35 human tissues. PLOS Comput Biol 11(5) 10.1371/journal.pcbi.1004220 PMC4430528 25970446

[CR70] Pitt M, Chan D, Kohn R (2006). Efficient bayesian inference for gaussian copula regression models. Biometrika.

[CR71] Regazzini E, Lijoi A, Prünster I (2003). Distributional results for means of random measures with independent increments. The Ann Stat.

[CR72] Roberts P, Der C (2007). Targeting the raf-mek-erk mitogen-activated protein kinase cascade for the treatment of cancer. Oncogene.

[CR73] Rothman AJ, Levina E, Zhu J (2010). Sparse multivariate regression with covariance estimation. J Comput Graph Stat.

[CR74] Roverato A (2000). Cholesky decomposition of a hyper-inverse Wishart matrix. Biometrika.

[CR75] Saegusa T, Shojaie A (2016). Joint estimation of precision matrices in heterogeneous populations. Electron J Stat.

[CR76] Scott J, Berger J (2010). Bayes and empirical-Bayes mutliplicity adjustment in the variable-selection problem. Ann Stat.

[CR77] Scott J, Carvalho C (2008). Feature-inclusion stochastic search for gaussian graphical models. J Comput Graph Stat.

[CR78] Shaddox E, Stingo FC, Peterson CB, Jacobson S, Cruickshank-Quinn C, Kechris K, et al. (2018) A Bayesian approach for learning gene networks underlying disease severity in COPD. Statistics in Biosciences pp 1–27 10.1007/s12561-016-9176-6 PMC8078135 33912251

[CR79] Shaddox E, Peterson CB, Stingo FC, Hanania NA, Cruickshank-Quinn C, Kechris K, Bowler R, Vannucci M (2020). Bayesian inference of networks across multiple sample groups and data types. Biostatistics.

[CR80] Shojaie A, Michailidis G (2010) Penalized principal component regression on graphs for analysis of subnetworks. In: Advances in Neural Information Processing Systems, pp 2155–2163

[CR81] Silke J, Brink R (2010). Regulation of tnfrsf and innate immune signalling complexes by trafs and ciaps. Cell Death Diff.

[CR82] Spirtes P, Glymour C, Scheines R (2000) Causation, prediction, and search, vol 81. The MIT Press

[CR83] Stingo F, Marchetti GM (2015). Efficient local updates for undirected graphical models. Stat Comput.

[CR84] Stingo F, Chen Y, Vannucci M, Barrier M, Mirkes P (2010). A Bayesian graphical modeling approach to microrna regulatory network inference. Ann Appl Stat.

[CR85] Stingo F, Chen Y, Tadesse M, Vannucci M (2011). Incorporating biological information into linear models: a Bayesian approach to the selection of pathways and genes. Ann Appl Stat.

[CR86] Tan L, Jasra A, De Iorio M, Ebbels T (2017). Bayesian inference for multiple Gaussian graphical models with application to metabolic association networks. The Ann Appl Stat.

[CR87] Telesca D, Mueller P, Kornblau S, Suchard M, Ji Y (2012). Modeling protein expression and protein signaling pathways. J Am Stat Assoc.

[CR88] Van Dyk D, Park T (2008). Partially collapsed gibbs samplers: theory and methods. J Am Stat Associ.

[CR89] Wang H (2012). Bayesian graphical lasso models and efficient posterior computation. Bayesian Anal.

[CR90] Wang H (2015). Scaling it up: stochastic search structure learning in graphical models. Bayesian Anal.

[CR91] Wang H, Li Z (2012). Efficient gaussian graphical model determination under G-Wishart prior distributions. Electron J Stat.

[CR92] Warnick R, Guindani M, Erhardt EB, Allen EA, Calhoun VD, Vannucci M (2018). A Bayesian approach for estimating dynamic functional network connectivity in fMRI data. J Am Stat Assoc.

[CR93] Williams DR, Rast P, Pericchi L, Mulder J (2019) Comparing gaussian graphical models with the posterior predictive distribution and bayesian model selection 10.1037/met0000254 PMC8572134 32077709

[CR94] Yajima M, Telesca D, Ji Y, Müller P (2014). Detecting differential patterns of interaction in molecular pathways. Biostatistics.

[CR95] Yin J, Li H (2011). A sparse conditional Gaussian graphical model for analysis of genetical genomics data. The Ann Appl Stat.

[CR96] Yuan M, Lin Y (2007). Model selection and estimation in the Gaussian graphical model. Biometrika.

[CR97] Zhou S, Lafferty J, Wasserman L (2010). Time varying undirected graphs. Mach Learn.

[CR98] Zhu Y, Shen X, Pan W (2014). Structural pursuit over multiple undirected graphs. J Am Stat Assoc.

